# Toward gene therapy of Laron syndrome

**DOI:** 10.1038/s41434-022-00329-2

**Published:** 2022-03-14

**Authors:** Haim Werner

**Affiliations:** Department of Human Molecular Genetics and Biochemistry, Sackler School of Medicine, Tel Aviv University, Tel Aviv, 69978 Israel

**Keywords:** Endocrine system and metabolic diseases, Molecular biology

The growth hormone (GH)-insulin-like growth factor-1 (IGF1) endocrine axis has a fundamental role in growth and development throughout life [1–3]. As originally postulated by Salmon and Daughaday in the mid-1950s, most of the biological actions of GH are mediated by a liver-produced peptide initially termed *somatomedin* and, subsequently, IGF1 [4]. IGF1 is structurally and evolutionarily related to the insulin molecule. IGF1 developed early in evolution, probably as a regulator of cellular proliferation in relation to nutrient availability. Prenatal IGF1 expression is GH-independent and becomes GH-dependent shortly before birth. Hepatic IGF1 biosynthesis remains dependent on hypophysial GH secretion during postnatal life.

Growth retardation in infants is multifactorial, although a large proportion of the cases remain *idiopathic* because no specific (genetic or other) defect can be identified [5, 6]. Congenital IGF1 deficiencies are characterized by low serum IGF1 but normal to elevated GH production [7]. These conditions result from:

1. GH releasing hormone-receptor (*GHRH-R*) defect;
2. *GH* gene deletion (isolated GH deficiency, IGHD);
3. GH receptor (*GHR*) gene deletion or mutation (Laron syndrome, LS);
4. *IGF1* gene deletion or IGF1 receptor (*IGF1R)* gene defect.

Additional conditions leading to congenital IGF1 deficiency include defective post-GHR signaling (e.g., STAT5 defect) as well as a number of disorders associated with reduced IGF1 stability or availability (e.g., acid-labile subunit, ALS, mutation) [8, 9].

Laron syndrome, or primary GH insensitivity (*OMIM#262500*), is an autosomal recessive disease caused by mutations in the *GHR* gene, leading to GH resistance and dwarfism. LS constitutes the best-characterized entity under the spectrum of the congenital IGF1 deficiencies. Clinical, genetic, and biochemical analyses of the disease conducted over the past sixty years have had a huge impact on our current understanding of GH-IGF1 pathophysiology [10, 11]. The disease was originally identified in the late 1950s in Jewish patients of Yemenite origin. Following the first report in 1966, LS patients of various ethnic origins were identified in different regions of the world [12, 13]. Of interest, most of the patients were of Mediterranean, Mid-Eastern or South Asian origin, including a large cohort in Ecuador [14]. According to most estimates, the approximate number of diagnosed LS patients worldwide ranges between 400 and 500 individuals. There is wide consensus, however, that many more patients remain undiagnosed.

**Fig. 1:**
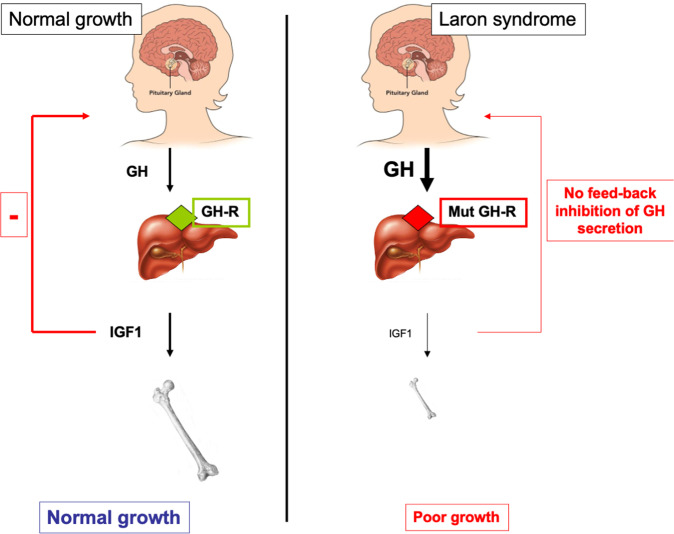
Schematic representation of the GH-IGF1 axis in health and in LS patients. Pituitary-produced GH leads to IGF1 secretion from the liver, with ensuing bone elongation and longitudinal growth (left panel). As a result of a *GHR* mutation in LS patients, the liver (and, most probably, also other extrahepatic tissues) is no longer able to produce IGF1 at physiological levels (right panel). Abrogation of IGF1 production leads to impaired growth and inadequate negative feedback at the pituitary gland, leading to high circulating GH levels (Figure adapted from Werner et al. [18]).

The distinctive features of LS include short stature (−4 to −10 SDS below median height), characteristic facial features, reduced head circumference, obesity, acromicria (i.e., smallness of the extremities), high basal serum GH, low to undetectable serum IGF1, and a lack of response to the administration of exogenous GH (Table 1) [10]. The identification of an exon deletion at the *GHR* gene as the molecular defect underlying LS etiology was reported in 1989 [15]. As a result of the *GHR* mutation there is a drastic reduction in IGF1 biosynthesis in the liver and, probably, other extrahepatic tissues, with ensuing dwarfism (Fig. 1). Lack of negative feedback at the pituitary level by the very low circulating IGF1 concentrations results in high GH levels, sometimes in the acromegaly range. Several *GHR* anomalies have been identified, including exon deletions and nonsense, frame-shift, and missense mutations. Despite the variability in the mutations observed, the phenotypic consequences are remarkably similar, i.e., dwarfism, lack of GH signaling and undetectable, or extremely low, IGF1 values.

**Table 1:** Typical features of Laron syndrome patients.

Short stature (−4 to −10 SDS below median height)
Obesity
Characteristic face features
Reduced head circumference
High basal serum GH
Low to undetectable serum IGF1 (unresponsive to exogenous GH)
Acromicria (small extremities)

The only treatment for LS is recombinant IGF1, available since 1986. One bolus injection reduced the serum levels of GH and glucose. Long-term IGF1 treatment using a single daily dose of 150–220 µg/kg body weight, given with the largest meal, resulted in a fast catch-up growth in head circumference, denoting brain growth, and a slower catch-up in linear growth, in comparison to that observed in GH-treated GH deficient children. In the first year of treatment, the growth velocity of LS children is typically ~8 cm/yr compared to 10-12 cm/yr in GH deficient children. After a decrease in body adiposity in the first months of IGF1 treatment, a progressive increase in obesity usually occurs [16]. In addition to obesity, authors reported other adverse effects such as hypoglycemia, transitory papilledema, headache, and swelling of lymphoid glands and spleen.

The paper “*First use of gene therapy to treat growth hormone resistant dwarfism in a mouse model*” by Sia et al. describes the development of a gene therapy approach in a mouse model of LS [17]. Authors employed a hepatocyte-specific adeno-associated virus expressing the mouse GHR (mGHR) gene in GHR deficient (GHR−/−) Laron dwarf mice. Authors formulated the following questions:

1. Would expression of GHR in the liver induce expression and secretion of IGF1?
2. What about IGFBP3 expression and IGF1 half-life?
3. And what about ALS levels and IGF1 stability?

The authors report that liver expression of mGHR significantly increased body weight and length of Laron dwarf mice. In addition, viral mGHR transfer enhanced IGF1, ALS, and IGFBP3 levels. These hormones induced a subsequent increase in femur length and organ (spleen, lung, heart) weights. No increase, however, was noticed in brain weight. The authors analyze the impact of restoration of GH signaling on endocrine and phenotypic parameters and discuss the main differences between IGF1 injections in LS patients and the novel gene transfer approach. In summary, Sia et al. provide proof-of-concept that gene therapy in Laron dwarf mice might have an efficacy similar to, or even better than, IGF1 treatment in patients. Furthermore, the data suggest that, eventually, this type of gene therapy approach may be useful for the treatment of LS in humans.
